# Acquisition Risk Factors of the SCC*mec* IX-Methicillin-Resistant *Staphylococcus aureus* in Swine Production Personnel in Chiang Mai and Lamphun Provinces, Thailand

**DOI:** 10.3390/antibiotics9100651

**Published:** 2020-09-29

**Authors:** Peerapat Rongsanam, Terdsak Yano, Wuttipong Yokart, Panuwat Yamsakul, Suweera Sutammeng, Ratchadaporn Udpaun, Duangporn Pichpol, Decha Tamdee, Usanee Anukool

**Affiliations:** 1 Division of Clinical Microbiology, Department of Medical Technology, Faculty of Associated Medical Sciences, Chiang Mai University, Chiang Mai 50200, Thailand; peerapat_rongsanam@cmu.ac.th (P.R.); gsrgrp2017@gmail.com (S.S.); ratchadaudp@hotmail.com (R.U.); 2 Department of Food Animal Clinic, Faculty of Veterinary Medicine, Chiang Mai University, Chiang Mai 50100, Thailand; terdsak.yano@cmu.ac.th (T.Y.); panuwat.y@cmu.ac.th (P.Y.); 3 Division of Clinical Microscopy, Department of Medical Technology, Faculty of Associated Medical Sciences, Chiang Mai University, Chiang Mai 50200, Thailand; Wuttipong.yokart@gmail.com; 4 Department of Veterinary Biosciences and Veterinary Public Health, Faculty of Veterinary Medicine, Chiang Mai University, Chiang Mai 50100, Thailand; duangporn.p@cmu.ac.th; 5 Department of Public Health Nursing, Faculty of Nursing, Chiang Mai University, Chiang Mai 50200, Thailand; decha.t@cmu.ac.th; 6 Infectious Diseases Research Unit (IDRU), Faculty of Associated Medical Sciences, Chiang Mai University, Muang District, Chiang Mai 50200, Thailand

**Keywords:** MRSA, *Staphylococcus aureus*, staphylococcal cassette chromosome *mec* (SCC*mec*), antimicrobial resistance, pig farming, acquisition risk factors

## Abstract

Methicillin-resistant *Staphylococcus aureus* (MRSA) harboring the type-IX staphylococcal cassette chromosome *mec* (SCC*mec*) has been found in pigs and humans in Northern Thailand. However, knowledge of the prevalence and acquisition risk factors of this MRSA strain among swine production personnel (SPP) are needed. The nasal swab samples and data were collected from 202 voluntary SPP and 31 swine farms in Chiang Mai and Lamphun Provinces, Thailand in 2017. MRSA were screened and identified using mannitol salt agar, biochemical and antimicrobial susceptibility testing, multiplex PCR, and the SCC*mec* typing. The prevalence of MRSA was 7.9% (16/202) and 19.3% (6/31) among SPP and swine farms. All isolates were multidrug-resistant, and 55 of 59 isolates (93%) contained the type-IX SCC*mec* element. Data analysis indicated that education, working time, contact frequency, working solely with swine production, and personal hygiene were significantly related to MRSA acquisition (*p* < 0.05). The multivariate analysis revealed that pig farming experience, working days, and showering were good predictors for MRSA carriage among SPP (area under the curve (AUC) = 0.84). The biosecurity protocols and tetracycline use were significantly associated with MRSA detection in pig farms (*p* < 0.05). Hence, the active surveillance of MRSA and further development of local/national intervention for MRSA control are essential.

## 1. Introduction

Methicillin-resistant *Staphylococcus aureus* (MRSA) is resistant to all beta-lactam antibiotics except ceftaroline. The hospital-associated (HA-) MRSA is typically resistant to multiple classes of antibiotics; thus, infections caused by MRSA usually result in prolonged hospitalization, extensive treatment, and a high economic burden. MRSA was also reported in patients who do not have common healthcare risk factors (e.g., previous surgery or history of hospital admission) in the late 1980s, and it was categorized as community-associated (CA-) MRSA [1,2]. In 2005, previous studies in France and the Netherlands provided evidence of a novel lineage of MRSA in pigs and pig farmers, clonal complex (CC) 398, which is now recognized as the livestock-associated MRSA (LA-MRSA) [3,4]. Since pigs have been postulated as a reservoir of LA-MRSA, the swine production personnel (SPP) were at risk for the occupational-associated exposure of MRSA. Several studies demonstrated the prevalence and MRSA-associated risk factors among SPP, including swine farm workers and slaughterhouse workers [5,6,7]. It was found that contact with pigs and the number of workers having contact with pigs were risk factors associated with MRSA carriage in pig farm workers [7]. In Thailand, LA-MRSA was reported in pigs and pork with a wide range of prevalence, from 0.63–50% [8,9,10,11]. However, knowledge about the prevalence and risk factors for the acquisition of MRSA in farm owners, veterinarians, animal husbandmen, and, also, veterinary and animal sciences students is still lacking.

Typing of the staphylococcal cassette chromosome *mec* (SCC*mec*) is based on the combination of the *mec* and *ccr* gene complexes, the key components of SCC*mec* element conferring methicillin resistance in staphylococci [12]. To date, SCC*mec* types I to XIII have been approved by the International Working Group on the Classification of Staphylococcal Cassette Chromosome Elements (IWG-SCC) [13]. While SCC*mec* I–III are commonly found in HA-MRSA, the SCC*mec* IV–XIII are usually detected in CA-MRSA and LA-MRSA [14]. The classification of SCC*mec* is, therefore, important for the investigation of the epidemiological background of MRSA clones. In 2011, MRSA harboring *ccrA1B1* and *mec* C2, presently classified into the type-IX SCC*mec*, was firstly reported in pigs in Thailand [8,9,10,11]. Since then, several studies have reported the detection of the ST9 MRSA clone harboring the type-IX SCC*mec* in pigs and pork, thus far only found in Thailand [8,9,10,11]. Not only in swine production, SCC*mec* IX-MRSA was reported in an outpatient of a hospital in Northeastern Thailand [15]. The results of pulse-field gel electrophoresis showed related patterns between MRSA strains from a patient, farm workers, and pigs in the same area [11]. These data suggested the presence and presumable spread of a unique LA-MRSA clone in Thailand. The ST9-SCC*mec* IX-MRSA clone probably has spread among the livestock, community, and hospitals. Though data of the prevalence of SCC*mec* IX-MRSA among SPP in Northern Thailand is still limited, a study reported a prevalence of SCC*mec* IV-MRSA in pig farm workers in Chiang Mai-Lamphun Province at 2.53% since 2014 [16]. In addition, there was no information about the risk factors of MRSA carriage among veterinarians, animal husbandmen, and veterinary and animal sciences students in Thailand. Thus, the purposes of this study are to investigate a more recent situation of LA-MRSA in SPP in Chiang Mai and Lamphun Provinces, Thailand and to elucidate the risk factors associated with MRSA carriage in SPP. The data achieved are expected to be beneficial for the further development of a local and national guideline to prevent and control LA-MRSA spread and infection in Thailand.

## 2. Results

### 2.1. MRSA Carriage Rate in Swine Production Personnel and Pig Farms

**Figure 1:**
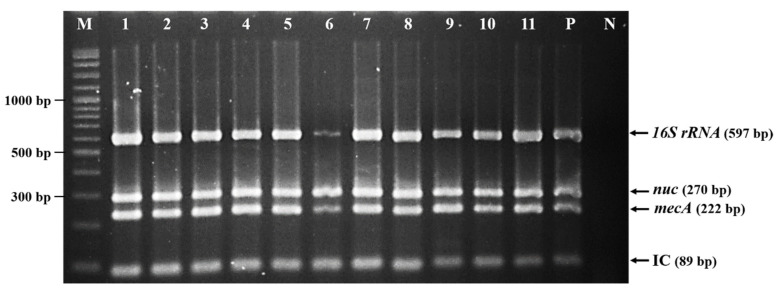
Gel electrophoresis of multiplex PCR for confirmation of methicillin-resistant *Staphylococcus aureus* (MRSA) isolates by detection of the staphylococcal 16S ribosomal RNA (*16S rRNA*), the *S. aureus*-specific thermonuclease (*nuc*), and the penicillin-binding protein 2a-encoding (*mecA*) genes. Phocid herpesvirus type 1 (PhHV-1) plasmid DNA was used as an internal control (IC). Lane M is a 100-bp DNA marker; lanes 1–11 are MRSA isolates X2-1, X2-2, X2-3, X2-4, X2-5, X3-1, X3-2, X3-3, X3-4, X3-5, and X3-6, respectively; lane P is the positive control (*S. aureus* ATCC 43300); and lane N is the negative control (sterile distilled water).

In total, 997 bacterial isolates from nasal swab samples of 202 SPP (Table S1) with a typical *S. aureus* colonial morphology (yellow, round, creamy, and sharp border), Gram-positive, and catalase-positive were selected from the oxacillin resistance screening agar plates. Among these, 220 isolates from 63 SPP were identified as *S. aureus* using biochemical tests. The cefoxitin disk test and multiplex PCR at last confirmed the MRSA phenotype and genotype of 59 *S. aureus* isolates from 16 SPP. Figure 1 showed the multiplex PCR results of 11 MRSA isolates. The MRSA carriage rate among SPP was calculated at 7.9% (16/202). The prevalence of MRSA carriage in swine farm owners, veterinarians or animal husbandmen, swine farm workers, and veterinary or animal sciences students were 13.3% (4/30), 11.8% (2/17), 9% (9/100), and 1.8% (1/55), respectively. There were six out of 31 participated swine farms that were positive for MRSA detection, which accounted for 19.3%.

### 2.2. Antimicrobial Resistance of MRSA Isolates

**Figure 2:**
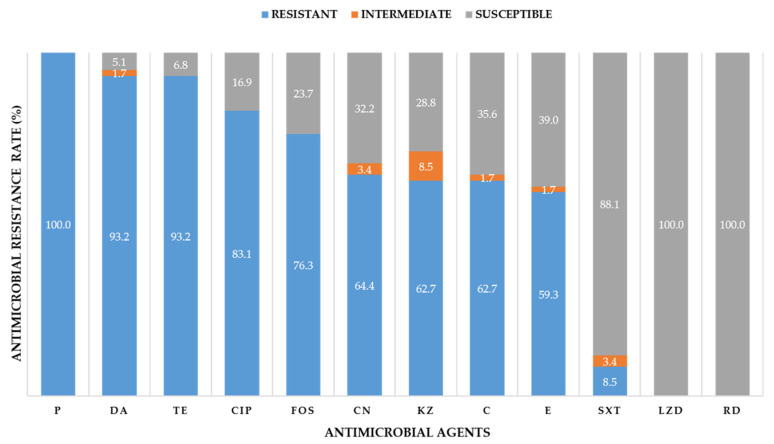
Antimicrobial resistance rate of 59 MRSA isolates from swine production personnel (SPP) tested against 12 antimicrobial agents: P, penicillin; DA, clindamycin; TE, tetracycline; CIP, ciprofloxacin; FOS, fosfomycin; CN, gentamycin; KZ, cefazolin; C, chloramphenicol; E, erythromycin; SXT, trimethoprim-sulfamethoxazole; LZD, linezolid; and RD, rifampicin.

The results of antimicrobial susceptibility testing are shown in Figure 2. All 59 MRSA isolates were resistant to penicillin but susceptible to linezolid and rifampicin. Most of all the 59 isolates were resistant to clindamycin, tetracycline, and ciprofloxacin at 93.2%, 93.2%, and 83.1%, respectively. Whereas 76.3%, 64.4%, 62.7%, 62.7%, and 59.3% of isolates were resistant to fosfomycin, gentamycin, cefazolin, chloramphenicol, and erythromycin, respectively, 8.5% were resistant to trimethoprim-sulfamethoxazole. In addition, 16 representative MRSA isolates from 16 nasal swab samples were susceptible to vancomycin (data not shown). All MRSA isolates showed multidrug-resistant (MDR) phenotypes. Approximately 93.2% of MRSA isolates were resistant to at least five antibiotic classes (Table S2). The maximum antibiotics classes that MRSA were resistant to was 10 out of 12 antimicrobial agents tested. The antimicrobial resistance profiles of MRSA isolates were highly diverse. They were classified into 19 different antimicrobial resistance patterns (I–XIX) (Table 1).

### 2.3. SCCmec Typing

Among the 59 MRSA isolates, 55 (93.2%) isolates were classified as SCC*mec* IX-MRSA, the livestock-related strain, except four isolates: one SCC*mec*-IV (1.7%) and three untypeable MRSA strains (5.1%). The SCC*mec* IX-MRSA strains were detected in all groups of SPP, including four farm owners, eight pig farm workers, two animal husbandmen, and one veterinary student. An SCC*mec* IV-MRSA and three untypeable MRSA strains were found in the farm workers.

### 2.4. Risk Factors of MRSA Detection in SPP and a Swine Farm

Most of the volunteers were male (113 vs. 89), with the mean age of 35.4 years old, ranging from 19 to 70 years old. The demographic characteristics and potential risk factors of MRSA carriage among the participant SPP are shown in Table 2. The level of education, working time in a farm, frequency of contact with pigs, good personal hygiene like changing work clothes before leaving the farm and showering after work, and working solely with swine production appeared to be significantly associated with MRSA detection among SPP (*p* < 0.05). After performing a multivariate analysis, the selected regression model with the lowest Akaike’s information criterion (AIC) value showed that the accurate predictors for MRSA carriage were the experience of working with pigs (adjusted odds ratio (OR) 0.92, 95% confidence interval (CI) 0.83–1.03, *p* = 0.141), working days per week (adjusted OR 4.2, 95% CI 0.98–18.05, *p* = 0.053), and showering after work (adjusted OR 0.14, 95% CI 0.04–0.49, *p* = 0.002). The receiver operating characteristic (ROC) curve with the area under the curve (AUC) of 0.84 was generated by using the R statistical package.

The data of 23 farms out of 31 participated farms were collected and analyzed. The demographic characteristics and potential risk factors of MRSA detection among 23 swine farms are shown in Table 3. The statistical analysis revealed there were several factors significantly associated with the presence of MRSA in the swine farm, including the total number of staff, the number of farm workers, the total number of pigs, the number of nursery pigs, long duration of disease outbreak in the farm, implementation of the appropriate method for personal and vehicle disinfection, regular water quality check, and the usage of tetracycline (*p* < 0.05).

## 3. Discussion

LA-MRSA has emerged in many parts of the world, especially in the region with a high density of swine production [3]. The SPP and the individuals with frequent contact with pigs were at risk for MRSA colonization. These people may be the source of MRSA transmission in households and the community [17]. This study showed that the MRSA carriage rates among SPP and swine farms in Chiang Mai and Lamphun Provinces, Thailand are increasing. The rates of MRSA in SPP and pig farms of 7.9% and 19.3% were observed in this study, while the prevalence rates of MRSA among swine farm workers and pig farms in 2014 were 2.53% and 9.61% [16].

Individuals working with pigs were at risk for the occupational acquisition or contamination of MRSA [7]. In this study, the highest carriage rate of MRSA was found in swine farm owners, followed by veterinarians or animal husbandmen, farm workers, and veterinary or animal sciences students. However, the differences in MRSA carriage rates between SPP groups were not statistically significant. The high rate of MRSA carriage found in the farm owners may be caused by the fact that 94% (16/17) of MRSA-positive farm owners also routinely worked in the swine farm (data not shown). The farm owners, farm workers, and veterinarians or animal husbandmen who routinely work with pigs and spend more time in pig farms might have a higher possibility for MRSA acquisition compared to veterinary or animal sciences students. However, occasional visits and practices at swine farms possibly caused contamination with MRSA among veterinary or animal sciences students. The study in Denmark showed that the exposure to airborne MRSA in the farm was associated with nasal MRSA carriage among volunteers visiting the swine farm [18]. A recent study also found that five of six swine farms in Denmark were positive for MRSA in airborne dust samples, with a half-life of five years, suggesting that dust might be the important transmission vehicle for MRSA in the farms [19].

The data obtained in this study indicated that eight animal antibiotics (penicillin, cephalosporin, tetracycline, macrolide, aminoglycoside, fluoroquinolone, trimethoprim-sulfamethoxazole, and colistin) were used for both the treatment and prevention of infectious diseases. The resistance rates of MRSA were found to be correlated with the usage of antibiotics in each farm (data not shown). Additionally, the frequency of antibiotic use in MRSA-positive swine farms (n = 4) were 100% for penicillins and macrolides; 75% for tetracyclines and quinolones; and 25% for aminoglycosides, trimethoprim-sulfamethoxazole, and colistin. This corresponds to the high resistance rates found for penicillin, clindamycin, tetracycline, ciprofloxacin, gentamycin, and erythromycin (Figure 2). However, the relationship between the frequency of antibiotic use and the frequency of MDR MRSA strains could not be statistically analyzed and concluded due to insufficient data. The high rates of resistance found in MRSA isolates from SPP to non-beta-lactam antibiotics, clindamycin (93.2%), tetracycline (93.2%), and ciprofloxacin (83.1%) were in concordance with several studies of MRSA in swine production in Thailand. An observational study in Chiang Mai and Lamphun Provinces reported tetracycline and clindamycin resistance in all 13 MRSA isolates from pigs, pig farm workers, and farm environments [16]. The studies in Northern, Northeastern, and Central Thailand found that almost all MRSA isolates from pigs and swine farm workers were resistant to tetracycline, clindamycin, and ciprofloxacin [9,11,20]. The antibiotics such as tetracycline, lincomycin, and amoxicillin were widely used in swine production, which can cause the development of antimicrobial resistance in the bacteria in pig intestines [21]. The tetracycline resistance gene, *tet(M)*, and tetracycline resistance phenotype were suggested as one of the markers for LA-MRSA [22,23,24,25]. Clindamycin, a lincosamide antibiotic, is important for the treatment of bacterial infections in pigs [26]. The resistance mechanisms to this antibiotic, including ribosomal methylation by *erm* (erythromycin ribosome methylase) genes and the *vgaALC* gene encoding resistance to lincosamide antibiotics, were reported [27,28]. Ciprofloxacin is one of the fluoroquinolone antibiotics used for the treatment of staphylococcal infection [29]. The fluoroquinolone resistance mechanism is based on the alteration of bacterial DNA gyrase and DNA topoisomerase IV, especially by the mutation of the quinolone resistance-determining regions (QRDR) of *gyrA* and *parC* genes [30,31]. To confirm their resistance mechanisms, further investigation of the drug resistance determinants associated with these drug resistance phenotypes is necessary.

In this study, the predominant strain of MRSA found in SPP in Chiang Mai and Lamphun Provinces, Thailand, was SCC*mec* IX-MRSA. In 2014, none of the SCC*mec* IX-MRSA but only the ST9-SCC*mec* IV-MRSA were found in the swine farm workers, pigs, and farm environment in Chiang Mai and Lamphun Provinces [16]. However, the ST9-SCC*mec* IX-MRSA strains appeared to be associated with swine production in Thailand. Several investigators reported the detection of this MRSA clone in pigs, pork, and veterinarians in Thailand [8,9,10,11,16]. Moreover, there were the reports of SCC*mec* IX-MRSA strains in patients at hospitals in Khon Kaen Province, Thailand [11]. These findings highlighted the worrisome situation of MRSA dissemination from livestock to the community and hospital. This study confirmed a high prevalence of the livestock-related SCC*mec* IX-MRSA strains among SPP. Nevertheless, further investigation using sequence-based molecular typing methods, such as multi-locus sequence typing (MLST) and staphylococcal protein A (*spa*) typing, is needed to provide insight into their molecular epidemiology. The additional study of the MRSA isolates from pigs and farm environments of the MRSA-positive swine farms is especially intriguing for a better understanding of the acquisition and transmission of MRSA between pigs, SPP, and farm environments.

Among four non-SCC*mec* IX MRSA isolates reported in this study, three isolates: Q1-1, Q1-2, and Q1-5 were isolated from a farm worker (Table S1). Isolate Q1-5 was classified as a SCC*mec* IV-MRSA, which was one of the community-related SCC*mec* types. However, two isolates were untypeable. Isolate Q1-1 possessed *ccr* 2, but the *mec* class could not be identified, because the targeted *mec* sequences were not amplified by the described method [32]. Neither the *ccr* type nor the *mec* class could be identified for isolate Q1-2 due to no amplification of the targeted sequences. The last untypeable isolate, X3-6, was detected in another farm worker (Table S1). It carried a class C-*mec*, but the amplicons of the targeted *ccr* sequences were not detected. The combination of the *mec* and *ccr* gene complexes determines the SCC*mec* type, while the J1 region is used for subtyping [12]. However, genetic rearrangement of the SCC*mec* element can result in novel elements, variants of existing SCC*mec* elements, and composite elements, hence complicating the nomenclature of SCC*mec* elements [13]. According to the method used in this study, the SCC*mec* typing of these MRSA strains may be limited to only SCC*mec* types I–VI, VIII, and IX. An in silico characterization of the SCC*mec* element from *S. aureus* whole-genome sequencing data such as SCCmecFinder might be useful for the classification of these untypeable MRSA strains [13].

The analysis of data collected in 2017 revealed many factors associated with MRSA occurrence in SPP and swine farms comprised of education, working time, contact frequency, working solely with swine production, personal hygiene, and the number of workers and pigs, the biosecurity protocols, and the use of tetracycline in the farms. However, a follow-up study collecting more recent data would help address whether there is a change of the MRSA acquisition risk factors and if the discovered risk factors reflect the changes in the MRSA populations. Therefore, further research is essential to gain a better understanding of the presence of MRSA in swine farms, as well as to provide the baseline data necessary for the development of local or national interventions and guidelines in Thailand.

The study of Sahibzada et al., 2018 demonstrated that the number of hours that individuals had contact with pigs appeared to be a significant factor for MRSA carriage in farm workers [7]. In this study, the working time and frequency of contact with pigs were potential factors for MRSA acquisition. However, the history of direct contact with pigs was not significantly associated with MRSA detection (*p* = 1.000). The reason for this may be that most data were obtained from the SPP who were occupationally exposed to swine production and have a history of direct contact with pigs. This study showed that working in farms that raised only pigs was a potential factor associated with MRSA acquisition. The results were discordant with other studies, which showed that LA-MRSA can be found in other livestock such as cattle and poultry and other companion animals such as horses and dogs [33,34]. The different outcomes could be explained by the differences in the prevalence and distribution of MRSA among livestock, the geographical areas, and the studied population. Hand hygiene and cleaning of the surface area affected MRSA reduction, especially in hospital settings [35]. In this study, hand washing and showering after work were significantly associated with MRSA carriage in SPP (*p* = 0.019 and *p* = 0.005, respectively). The results supported that personal hygiene was an effective practice and may reduce MRSA contamination while working in swine farms or other swine production areas. The multivariate analysis showed that experience working with pigs, working days per week, and showering after work were moderately accurate independent predictors for MRSA carriage among SPP, with AUC of 0.84. The 0.7 ≤ AUC < 0.9 indicate moderately accurate predictions, while 0.9 ≤ AUC < 1.0 indicate highly accurate predictions [36]. This was consistent with the study by van Cleef et al., 2014. The multivariate analysis showed that age, working time, giving birth assistance to sows, and wearing masks were significantly associated with persistent MRSA carriage among pig farmers [37].

Several studies revealed that the density of pigs in farms was associated with LA-MRSA acquisition [38,39,40]. The study in Denmark showed that living near pig farms was a risk factor for LA-MRSA carriage [41]. As a potential source of LA-MRSA, pigs may carry LA-MRSA strains and then spread the organism to the SPP or the farm environment [42,43]. The contaminated dust in the farms may play a role as the transmission vehicle of MRSA in farm environments [19]. Thus, a suitable method for personal and environmental cleaning was needed to reduce MRSA contamination. Notably, personal disinfection and the use of personal protective equipment such as work clothes, gloves, and masks are also important. The association between tetracycline usage and the presence of MRSA in swine farms was in concordance with the previous studies. They showed that the usage of tetracycline in weaner pigs affected the MRSA status of the farms [44]. Therefore, the usage of tetracycline should be avoided, and the appropriate use of all antimicrobial agents, especially the drug classes used for the treatment of both humans and animals, is highly important to prevent the increased colonization of MRSA. Besides, the use of certain disinfectants might affect the frequency of MDR MRSA strains. In Thailand, the disinfection agent generally used in dairy and swine farms for the effective killing of bacteria and viruses is glutaraldehyde [45]. The resistance to glutaraldehyde was reported in Gram-negative bacteria such as *Pseudomonas aeruginosa, Pseudomonas fluorescens*, and *Riemerella anatipestifer* [46,47]. However, the cross-resistance to antibiotic drugs affected by low-level exposure to glutaraldehyde has not been described in Gram-positive bacteria [48].

The results from this study suggested that SPP, pigs, and swine farm environments probably were the sources of MRSA infections. As a part of our study, the results of MRSA detection and risk factors associated with the MRSA carriage were communicated to all participants, both MRSA-positive and MRSA-negative SPP. The result report form was developed to explain the potential sources of MRSA acquisition and infection, along with the prevention guidelines to the participants, which will ensure their safe operations in swine farms, as well as a healthy daily routine. Notably, the data obtained indicated that all MRSA-negative farms followed the biosecurity recommendations of Thai Agricultural Standard, TAS 6403-2009: Good agricultural practices for pig farm (www.afcs.go.th). The disinfection methods such as the vehicle disinfection pond, disinfectant spraying houses, and disinfectant spraying machines were applied. In addition, physical barriers such as having a fence to separate the production area from residential areas were used by most MRSA-negative farms. These control measures and hygienic practices are applicable to nearly all swine farms and SPP in Thailand. However, the awareness and understanding of infection control and antimicrobial resistance among SPP, especially workers and owners of the small-holder swine farms, need to be strengthened.

## 4. Conclusions

In conclusion, this study revealed the relatively high prevalence of SCC*mec* IX-MRSA with MDR phenotypes among SPP in Chiang Mai and Lamphun Provinces, Thailand. The personal hygienic practice, suitable farm management, and appropriate antibiotics uses are highly recommended for the prevention and reduction of MRSA carriage via occupational exposure of the contaminated pigs and swine farm environments. The awareness of MRSA, active surveillance of MRSA in the swine production chain, and good agricultural practice for pig farms are crucial for the prevention of MRSA dissemination in swine farms and the community.

## 5. Materials and Methods

### 5.1. Ethics Approval

The ethics approval of this study was obtained from the ethics committee of Faculty of Associated Medical Sciences, Chiang Mai University (Approval code: AMSEC-598X-034) prior to the recruitment of all participants. All methods were performed following the relevant guidelines and regulations.

### 5.2. Data and Sample Collection

Two-hundred and two SPP and 31 swine farms in Chiang Mai and Lamphun Provinces voluntarily participated in this study (Table S1). The 202 SPP included 100 swine farm workers, 30 farm owners, 17 veterinarians or animal husbandmen, and 55 veterinary or animal sciences students. The developed questionnaire was validated and then used to collect the individual SPP and swine farm data. All volunteers were informed about the project information and voluntarily signed the consent forms before the data and nasal swab samples were collected. The data were collected from the participants in either the written format or oral interview.

### 5.3. Bacterial Isolation and Identification

All nasal swab samples were pre-enriched in brain heart infusion broth supplemented with 7% sodium chloride and incubated overnight at 35 ± 2 °C. Twenty microliters of enriched samples were then cultured on Mannitol salt agar supplemented with 2 and 4-µg/mL oxacillin and incubated overnight at 35 ± 2 °C. At least 2 single yellow colonies with round, creamy, and sharp borders were selected and identified by standard biochemical tests. *S. aureus* isolates were subjected to the cefoxitin (30 µg) diffusion test for MRSA detection according to the Clinical and Laboratory Standards Institute (CLSI) standard (M100-S27, 2017). The genomic DNA of MRSA isolates was extracted by the method described by a previous study with some modification and stored at −20 °C for further analysis [49]. MRSA genotypes were confirmed by the detection of *16S rRNA*, *nuc*, and *mecA* genes using the primers shown in Table 4. The phocid herpesvirus type 1 (PhHV-1) plasmid DNA was used as an internal control for multiplex PCR amplification. The reaction mixture contained 1 µL of DNA extract, 0.64 µM of 16S rRNA-F and 16S rRNA-R primers, 0.192 µM of nuc-F and nuc-R primers, 0.288 µM of mecA-F and mecA-R primers, 0.4 µM of PhHV-F and PhHV-R primers, 1X of i-Taq PCR Master mix Solution (iNtRON Biotechnology, Gyeonggi, Republic of Korea), and sterile distilled water to adjust a final volume of 25 µL. The PCR program included an initial denaturation step at 94 °C, 4 min, 35 cycles of denaturation at 94 °C, 30 s, annealing at 58 °C, 30 s, extension at 72 °C, 30 s, and a final extension step at 72 °C for 4 min. Sterile distilled water and DNA of *S. aureus* ATCC 43300 (MRSA) were included in each PCR run as negative and positive controls.

### 5.4. Antimicrobial Susceptibility Testing

To determine the antimicrobial resistance of MRSA isolates, the disk diffusion method was performed and interpreted according to the CLSI standard (M100-S27, 2017). The tested antimicrobial disks (Oxoid Ltd., Hampshire, UK) included penicillin (10 Units), erythromycin (15 µg), clindamycin (2 µg), trimethoprim-sulfamethoxazole (25 µg), ciprofloxacin (5 µg), chloramphenicol (30 µg), gentamicin (10 µg), rifampin (5 µg), tetracycline (30 µg), cephazolin (30 µg), fosfomycin (50 µg), and linezolid (30 µg).

### 5.5. SCCmec Typing

The SCC*mec* types of MRSA isolates were characterized by the multiplex PCR described earlier [32]. The M-PCR1 and M-PCR 2 were applied to classify five *ccr* types and *mec* classes A to C. The primers used in M-PCR 1 and M-PCR 2 are shown in Table 5. The reaction mixtures and conditions were the same as it was explained in the previous study [32], except that 2.5 U of Dream *Taq* polymerase (Thermo Fisher Scientific Baltics UAB, Vilnius, Lithuania) was used in place of 2.5-U Ex *Taq* polymerase (Takara Bio Inc., Kyoto, Japan). In consistence with the guidelines proposed by the IWG-SCC, the SCC*mec* was then classified by a combination of *ccr* types and *mec* classes (http://www.sccmec.org). The genomic DNA of four MRSA-typed strains, including epidemic MRSA (EMRSA)-8, N315, EMRSA-4, and EMRSA-10, were included in each round of PCR as the controls for the classification of types I to IV SCC*mec*, respectively.

### 5.6. Data Analysis

The individual SPP data, the swine farm data, and the MRSA detection were analyzed using the R statistical package [53]. The individual that was positive for MRSA detection was defined as a MRSA carrier. The swine farms that provided at least one MRSA carrier were defined as MRSA-positive farms. The association between MRSA carriage and variable factors were analyzed using the chi-square test, Fisher’s exact tests, or Student’s *t*-test. A *p*-value lower than 0.05 was considered statistically significant. Multivariate analysis for potential risk factors were analyzed using logistic regression analysis. Multivariate regression models were constructed using stepwise regression and the minimum Akaike’s information criterion was the criterion for exiting the model. A model with the lowest AIC, the most parsimonious fit, was selected. Multivariate regression model accuracy was determined using receiver operating characteristic (ROC) curves.

## Figures and Tables

**Table 1:** The antimicrobial resistance profiles of 59 methicillin-resistant *Staphylococcus aureus* (MRSA) isolates from swine production personnel (SPP).

Antimicrobial Resistance Profiles		No. of MRSA Isolates	Percentage (%)
I.	P-TE-CIP-DA-C-FOS-CN-KZ-E-SXT	1	1.69
II.	P-TE-CIP-DA-C-FOS-CN-KZ-E	5	8.47
III.	P-TE-CIP-DA-C-CN-KZ-E-SXT	2	3.39
IV.	P-TE-CIP-DA-C-FOS-CN-KZ	1	1.69
V.	P-TE-CIP-DA-C-FOS-CN-E	4	6.78
VI.	P-TE-CIP-DA-C-CN-KZ-E	2	3.39
VII.	P-TE-CIP-DA-C-CN-E-SXT	1	1.69
VIII.	P-TE-CIP-DA-CN-KZ-E	9	15.25
IX.	P-TE-CIP-C-CN-KZ-E	1	1.69
X.	P-TE-CIP-DA-C-FOS-CN	5	8.47
XI.	P-TE-CIP-DA-C-FOS-KZ	6	10.17
XII.	P-TE-CIP-DA-C-CN-KZ	5	8.47
XIII.	P-TE-CIP-DA-C-CN-E	1	1.69
XIV.	P-TE-CIP-DA-C-FOS	3	5.08
XV.	P-TE-DA-FOS-KZ-E	2	3.39
XVI.	P-TE-DA-FOS-E	7	11.86
XVII.	P-CIP-CN-KZ	1	1.69
XVIII.	P-CIP-KZ	2	3.39
XIX.	P-DA-FOS	1	1.69
Total		59	100

P, penicillin; DA, clindamycin; TE, tetracycline; CIP, ciprofloxacin; FOS, fosfomycin; CN, gentamycin; KZ, cefazolin; C, chloramphenicol; E, erythromycin; and SXT, trimethoprim-sulfamethoxazole.

**Table 2:** The demographic characteristics and potential risk factors of MRSA carriage among the participant swine production personnel.

Characteristics	Number	MRSA-Positive	*p*-Value
General information			
Age (years)	35.43 (19–70)	32.77	0.749
Gender			0.306
Male	113 (56%)	7 (6%)	
Female	89 (44%)	9 (10%)	
Education			0.031 *
None	30 (15%)	7 (23%)	
Primary school	33 (17%)	0 (0%)	
Grade 9	19 (10%)	2 (10%)	
High school	20 (10%)	1 (5%)	
Diploma	11 (6%)	0 (0%)	
Bachelor’s degree	73 (37%)	5 (7%)	
Postgraduate	13 (6%)	1 (8%)	
Occupation and pig contact frequency			
Role of SPP in farms			0.211
Farm owner	30 (15%)	4 (13%)	
Farm worker	100 (50%)	2 (9%)	
Veterinarian/animal husbandman	17 (8%)	9 (2%)	
Veterinary/animal sciences students	55 (27%)	1(12%)	
Experience of working with pigs (months)	9.28 (0–36)	6.23	0.091
Direct contact with pigs			1.000
Yes	187 (94%)	15 (8%)	
No	12 (6%)	1 (8%)	
Frequency of contact with pigs			0.013 *
High (≥24 days/month)	102 (57%)	15 (14%)	
Medium (9–23 days/month)	14 (8%)	0 (0%)	
Low (≤8 days/month)	62 (35%)	1 (2%)	
Number of working hours in a day (hours)	5.99 (1–15)	7.33	0.047 *
Number of working hours in a week (hours)	34.15 (1–105)	50.27	0.004 **
Number of working days in a week (days)	5.24 (1–7)	6.87	0.003 **
Raise livestock other than pigs			0.026 *
Yes	46 (23)	0 (0%)	
No	153 (77)	16 (10%)	
Personal hygiene			
Hand washing			1.000
Yes	193 (96%)	16 (8%)	
No	9 (4%)	0 (0%)	
Changing clothes before leaving the farm			0.019 *
Yes	130 (64%)	6 (5%)	
No	72 (36%)	10 (14%)	
Shower after work			0.005 **
Yes	162 (80%)	8 (5%)	
No	40 (10%)	8 (20%)	
Eating during work			0.190
Yes	164 (81%)	5 (7%)	
No	38 (19%)	11 (17%)	
Cleaning the equipment			1.000
Yes	183 (91%)	15 (8%)	
No	18 (9%)	1 (6%)	
History of medication			
Antimicrobial drugs use in the previous year			0.436
Yes	133 (66%)	4 (6%)	
No	68 (34%)	12 (9%)	
Type of antimicrobial drugs			0.739
Amoxicillin	27 (57%)	1 (4%)	
Amoxicillin/Clavulanic acid	1 (2%)	0 (0%)	
Cloxacillin	4 (8%)	0 (0%)	
Oxytetracycline	1 (2%)	0 (0%)	
Other	15 (31%)	2 (13%)	
Received drugs by			
Prescription			0.397
Yes	34 (39%)	1 (3%)	
No	53 (61%)	5 (9%)	
Self-buying from drugstores			0.339
Yes	24 (27%)	3 (12%)	
No	64 (73%)	3 (5%)	
Other ways			1.000
Yes	4 (4%)	0 (0%)	
No	84 (96%)	6 (7%)	

The *p*-value was based on Fisher’s exact test and Student’s *t*-test, and the comparison was between MRSA carriers and non-MRSA carriers: * *p*-value less than 0.05 and ** *p*-value less than 0.01. MRSA, methicillin-resistant *S. aureus* and SPP, swine production personnel.

**Table 3:** The demographic characteristics and potential risk factors associated with MRSA detection among the participant swine farms.

Characteristics	Number	MRSA-Positive	*p*-Value
General information			
No. of staff			
Veterinarian	0.13 (0–1)	0.20	0.649
Animal Husbandman	0.16 (0–4)	1.40	0.050
Owner	1.22 (0–5)	0.80	0.278
Worker	5.22 (0–79)	19.40	0.024 *
Other (Housekeepers)	0.91 (0–12)	2.80	0.064
Total no. of staff	8.09 (0–96)	24.60	0.028 *
No. of pigs			
Suckling pigs	36.01 (0–500)	20.00	0.549
Nursery pigs	367.00 (4–6038)	1421.60	0.0288 *
Starter pigs	88.61 (0–400)	130.00	0.434
Grower pigs	76.44 (0–400)	120.00	0.412
Finisher pigs	507.35 (0–9000)	1928.00	0.051
Boars	8.00 (0–92)	22.40	0.060
Sows	287.26 (4–4671)	1044.20	0.044
Total no. of pigs	1370.87 (20–19,801)	4686.20	0.036 *
Farm management			
Regular water quality check			0.048 *
Yes	18 (78%)	2 (11%)	
No	5 (22%)	3 (60%)	
Method for vehicle disinfection			0.045 *
Disinfection pond	2 (9%)	0 (0%)	
Disinfectant spraying house	10 (43%)	3 (38%)	
Other (Disinfectant spraying machine)	8 (35%)	2 (67%)	
None	3 (13%)	0 (0%)	
Methods for personal disinfection			0.002 **
Bathroom	2 (10%)	1 (100%)	
Boot disinfecting bath	1 (5%)	0 (0%)	
More than 1 method	10 (50%)	2 (100%)	
Other (i.e., changing boots)	2 (10%)	0 (0%)	
None	5 (25%)	0 (0%)	
History of disease outbreak			
Disease outbreak in the previous year			0.155
Yes	12 (52%)	1 (8%)	
No	11 (48%)	4 (36%)	
Duration since the outbreak started until ended (months)	3.19 (1–8)	8	0.024 *
Antimicrobial use			
Penicillin			0.554
Yes	18 (82%)	4 (22%)	
No	4 (18%)	0 (0%)	
Tetracycline			0.046 *
Yes	6 (27%)	3 (50%)	
No	16 (73%)	1 (6%)	
Macrolide			0.096
Yes	12 (54%)	4 (33%)	
No	10 (46%)	0 (0%)	
Aminoglycoside			1.000
Yes	8 (36%)	1 (12%)	
No	14 (64%)	3 (23%)	
Fluoroquinolone			0.616
Yes	13 (59%)	3 (23%)	
No	9 (41%)	1 (11%)	
Cephalosporin			1.000
Yes	1 (4%)	0 (0%)	
No	21 (96%)	4 (19%)	
Trimethoprim-sulfamethoxazole			0.338
Yes	2 (9%)	1 (50%)	
No	20 (91%)	3 (15%)	
Colistin			1.000
Yes	4 (18%)	1 (25%)	
No	18 (82%)	3 (17%)	

The *p*-value was based on Fisher’s exact test and Student’s *t*-test, and the comparison was between MRSA-positive and -negative swine farms: * *p*-value less than 0.05 and **p-value less than 0.01.

**Table 4:** Primers used in the multiplex PCR for confirmation of MRSA.

Primers	Sequence (5′→3′)	Target	Amplicon Size (bp)	References
16S rRNA-F	GCAAGCGTTATCCGGATTT	*16S rRNA*	597	[50]
16S rRNA-R	CTTAATGATGGCAACTAAGC			
nuc-F	GCGATTGATGGTGATACGGTT	*nuc*	270	[51]
nuc-R	AGCCAAGCCTTGACGAACTAAAGC			
mecA-F	GCAATCGCTAAAGAACTAAG	*mecA*	222	
mecA-R	GGGACCAACATAACCTAATA			
PhHV-F	GGGCGAATCACAGATTGAATC	PhHV-1	89	[52]
PhHV-R	GCGGTTCCAAACGTACCAA			

*16S rRNA*, the staphylococcal 16S ribosomal RNA; *nuc*, the *S. aureus*-specific thermonuclease; *mecA*, a gene encodes penicillin-binding protein 2a (PBP2a); and PhHV-1, the phocid herpesvirus type 1.

**Table 5:** Primers used for the characterization of the staphylococcal cassette chromosome *mec* (SCC*mec*) types.

Primers	Sequence (5′→3′)	Target	Amplicon Size (bp)	References
MPCR1 (amplify *ccr* types with *mecA*)				[32]
mA1	TGCTATCCACCCTCAAACAGG	*mecA*	286	
mA2	AACGTTGTAACCACCCCAAGA			
α1	AACCTATATCATCAATCAGTACGT	*ccrA1-ccrB1*	695	
α2	TAAAGGCATCAATGCACAAACACT	*ccrA2-ccrB2*	937	
α3	AGCTCAAAAGCAAGCAATAGAAT	*ccrA3-ccrB3*	1791	
βc	ATTGCCTTGATAATAGCCITCT			
α4.2	GTATCAATGCACCAGAACTT	*ccrA4-ccrB4*	1287	
β4.2	TTGCGACTCTCTTGGCGTTT			
γR	CCTTTATAGACTGGATTATTCAAAATAT	*ccrC*	518	
γF	CGTCTATTACAAGATGTTAAGGATAAT			
MPCR2 (amplify *mec* classes)				[32]
mI6	CATAACTTCCCATTCTGCAGATG	*mecA-mecI* *mecA*-IS*1272* *mecA*-IS*431*	1963 2827 804	
IS7	ATGCTTAATGATAGCATCCGAATG			
IS2	TGAGGTTATTCAGATATTTCGATGT			
mI7	ATATACCAAACCCGACAACTACA			

IS, insertion sequence; *ccr*, the *ccr* gene complex; *mec*, the *mec* gene complex; and *mecA*, a gene encodes penicillin-binding protein 2a (PBP2a).

## Supplementary Materials

The following are available online at https://www.mdpi.com/2079-6382/9/10/651/s1: Table S1: The sampling sites, locations, and sample codes of 202 swine production personnel (SPP). Table S2: The accumulated resistance of 59 MRSA isolates from swine production personnel (SPP).

## References

[1] NeVille-Swensen M., Clayton M. (2011). Outpatient management of community-associated methicillin-resistant *Staphylococcus aureus* skin and soft tissue infection. J. Pediatric Health Care.

[2] Meddles-Torres C., Hu S., Jurgens C. (2013). Changes in prescriptive practices in skin and soft tissue infections associated with the increased occurrence of community acquired methicillin resistant *Staphylococcus aureus*. J. Infect. Public Health.

[3] Voss A., Loeffen F., Bakker J., Klaassen C., Wulf M. (2005). Methicillin-resistant *Staphylococcus aureus* in pig farming. Emerg. Infect. Dis..

[4] Armand-Lefevre L., Ruimy R., Andremont A. (2005). Clonal comparison of *Staphylococcus aureus* isolates from healthy pig farmers, human controls, and pigs. Emerg. Infect. Dis..

[5] Wang X.L., Li L., Li S.M., Huang J.Y., Fan Y.P., Yao Z.J., Ye X.H., Chen S.D. (2017). Phenotypic and molecular characteristics of *Staphylococcus aureus* and methicillin-resistant *Staphylococcus aureus* in slaughterhouse pig-related workers and control workers in Guangdong Province, China. Epidemiol. Infect..

[6] Gilbert M.J., Bos M.E., Duim B., Urlings B.A., Heres L., Wagenaar J.A., Heederik D.J. (2012). Livestock-associated MRSA ST398 carriage in pig slaughterhouse workers related to quantitative environmental exposure. Occup. Environ. Med..

[7] Sahibzada S., Hernandez-Jover M., Jordan D., Thomson P.C., Heller J. (2018). Emergence of highly prevalent CA-MRSA ST93 as an occupational risk in people working on a pig farm in Australia. PLoS ONE.

[8] Anukool U., O’Neill C.E., Butr-Indr B., Hawkey P.M., Gaze W.H., Wellington E.M. (2011). Meticillin-resistant *Staphylococcus aureus* in pigs from Thailand. Int. J. Antimicrob. Agents.

[9] Larsen J., Imanishi M., Hinjoy S., Tharavichitkul P., Duangsong K., Davis M.F., Nelson K.E., Larsen A.R., Skov R.L. (2012). Methicillin-resistant *Staphylococcus aureus* ST9 in pigs in Thailand. PLoS ONE.

[10] Vestergaard M., Cavaco L.M., Sirichote P., Unahalekhaka A., Dangsakul W., Svendsen C.A., Aarestrup F.M., Hendriksen R.S. (2012). SCC*mec* type IX element in methicillin resistant *Staphylococcus aureus* spa type t337 (CC9) isolated from pigs and pork in Thailand. Front. Microbiol..

[11] Sinlapasorn S., Lulitanond A., Angkititrakul S., Chanawong A., Wilailuckana C., Tavichakorntrakool R., Chindawong K., Seelaget C., Krasaesom M., Chartchai S. (2015). SCC*mec* IX in meticillin-resistant *Staphylococcus aureus* and meticillin-resistant coagulase-negative staphylococci from pigs and workers at pig farms in Khon Kaen, Thailand. J. Med. Microbiol..

[12] International Working Group on the Classification of Staphylococcal Cassette Chromosome Elements (2009). Classification of staphylococcal cassette chromosome *mec* (SCC*mec*): Guidelines for reporting novel SCC*mec* elements. Antimicrob. Agents Chemother..

[13] Kaya H., Hasman H., Larsen J., Stegger M., Johannesen T.B., Allesoe R.L., Lemvigh C.K., Aarestrup F.M., Lund O., Larsen A.R. (2018). SCCmecFinder, a web-based tool for typing of staphylococcal cassette chromosome *mec* in *Staphylococcus aureus* using whole-genome sequence data. mSphere.

[14] Lakhundi S., Zhang K. (2018). Methicillin-resistant *Staphylococcus aureus*: Molecular characterization, evolution, and epidemiology. Clin. Microbiol. Rev..

[15] Lulitanond A., Ito T., Li S., Han X., Ma X.X., Engchanil C., Chanawong A., Wilailuckana C., Jiwakanon N., Hiramatsu K. (2013). ST9 MRSA strains carrying a variant of type IX SCC*mec* identified in the Thai community. BMC Infect. Dis..

[16] Patchanee P., Tadee P., Arjkumpa O., Love D., Chanachai K., Alter T., Hinjoy S., Tharavichitkul P. (2014). Occurrence and characterization of livestock-associated methicillin-resistant *Staphylococcus aureus* in pig industries of northern Thailand. J. Vet. Sci..

[17] Becker K., Ballhausen B., Kahl B.C., Kock R. (2017). The clinical impact of livestock-associated methicillin-resistant *Staphylococcus aureus* of the clonal complex 398 for humans. Vet. Microbiol..

[18] Angen O., Feld L., Larsen J., Rostgaard K., Skov R., Madsen A.M., Larsen A.R. (2017). Transmission of methicillin-resistant *Staphylococcus aureus* to human volunteers visiting a swine farm. Appl. Environ. Microbiol..

[19] Feld L., Bay H., Angen O., Larsen A.R., Madsen A.M. (2018). Survival of LA-MRSA in dust from swine farms. Ann. Work Expo. Health.

[20] Chanchaithong P., Perreten V., Am-In N., Lugsomya K., Tummaruk P., Prapasarakul N. (2019). Molecular characterization and antimicrobial resistance of livestock-associated methicillin-resistant *Staphylococcus aureus* isolates from pigs and swine workers in central Thailand. Microb. Drug Resist..

[21] Sun J., Li L., Liu B., Xia J., Liao X., Liu Y. (2014). Development of aminoglycoside and beta-lactamase resistance among intestinal microbiota of swine treated with lincomycin, chlortetracycline, and amoxicillin. Front. Microbiol..

[22] Rinsky J.L., Nadimpalli M., Wing S., Hall D., Baron D., Price L.B., Larsen J., Stegger M., Stewart J., Heaney C.D. (2013). Livestock-associated methicillin and multidrug resistant *Staphylococcus aureus* is present among industrial, not antibiotic-free livestock operation workers in North Carolina. PLoS ONE.

[23] McCarthy A.J., Witney A.A., Gould K.A., Moodley A., Guardabassi L., Voss A., Denis O., Broens E.M., Hinds J., Lindsay J.A. (2011). The distribution of mobile genetic elements (MGEs) in MRSA CC398 is associated with both host and country. Genome Biol. Evol..

[24] McCarthy A.J., van Wamel W., Vandendriessche S., Larsen J., Denis O., Garcia-Graells C., Uhlemann A.C., Lowy F.D., Skov R., Lindsay J.A. (2012). *Staphylococcus aureus* CC398 clade associated with human-to-human transmission. Appl. Environ. Microbiol..

[25] Ye X., Wang X., Fan Y., Peng Y., Li L., Li S., Huang J., Yao Z., Chen S. (2016). Genotypic and phenotypic markers of livestock-associated methicillin-resistant *Staphylococcus aureus* CC9 in humans. Appl. Environ. Microbiol..

[26] Pyorala S., Baptiste K.E., Catry B., van Duijkeren E., Greko C., Moreno M.A., Pomba M.C., Rantala M., Ruzauskas M., Sanders P. (2014). Macrolides and lincosamides in cattle and pigs: Use and development of antimicrobial resistance. Vet. J..

[27] Leclercq R. (2002). Mechanisms of resistance to macrolides and lincosamides: Nature of the resistance elements and their clinical implications. Clin. Infect. Dis..

[28] Lopes E., Conceicao T., Poirel L., de Lencastre H., Aires-de-Sousa M. (2019). Epidemiology and antimicrobial resistance of methicillin-resistant *Staphylococcus aureus* isolates colonizing pigs with different exposure to antibiotics. PLoS ONE.

[29] Gade N.D., Qazi M.S. (2013). Fluoroquinolone therapy in *Staphylococcus aureus* infections: Where do we stand?. J. Lab. Physicians.

[30] Hooper D.C., Jacoby G.A. (2015). Mechanisms of drug resistance: Quinolone resistance. Ann. N. Y. Acad. Sci..

[31] Yamada M., Yoshida J., Hatou S., Yoshida T., Minagawa Y. (2008). Mutations in the quinolone resistance determining region in Staphylococcus epidermidis recovered from conjunctiva and their association with susceptibility to various fluoroquinolones. Br. J. Ophthalmol..

[32] Kondo Y., Ito T., Ma X.X., Watanabe S., Kreiswirth B.N., Etienne J., Hiramatsu K. (2007). Combination of multiplex PCRs for staphylococcal cassette chromosome *mec* type assignment: Rapid identification system for *mec*, *ccr*, and major differences in junkyard regions. Antimicrob. Agents Chemother..

[33] Kock R., Harlizius J., Bressan N., Laerberg R., Wieler L.H., Witte W., Deurenberg R.H., Voss A., Becker K., Friedrich A.W. (2009). Prevalence and molecular characteristics of methicillin-resistant *Staphylococcus aureus* (MRSA) among pigs on German farms and import of livestock-related MRSA into hospitals. Eur. J. Clin. Microbiol. Infect. Dis..

[34] Aires-de-Sousa M. (2017). Methicillin-resistant *Staphylococcus aureus* among animals: Current overview. Clin. Microbiol. Infect..

[35] Marimuthu K., Pittet D., Harbarth S. (2014). The effect of improved hand hygiene on nosocomial MRSA control. Antimicrob. Resist. Infect. Control.

[36] Swets J.A. (1988). Measuring the accuracy of diagnostic systems. Science.

[37] van Cleef B.A., van Benthem B.H., Verkade E.J., van Rijen M., Kluytmans-van den Bergh M.F., Schouls L.M., Duim B., Wagenaar J.A., Graveland H., Bos M.E. (2014). Dynamics of methicillin-resistant *Staphylococcus aureus* and methicillin-susceptible *Staphylococcus aureus* carriage in pig farmers: A prospective cohort study. Clin. Microbiol. Infect..

[38] Feingold B.J., Silbergeld E.K., Curriero F.C., van Cleef B.A., Heck M.E., Kluytmans J.A. (2012). Livestock density as risk factor for livestock-associated methicillin-resistant *Staphylococcus aureus*, The Netherlands. Emerg. Infect. Dis..

[39] Schinasi L., Wing S., Augustino K.L., Ramsey K.M., Nobles D.L., Richardson D.B., Price L.B., Aziz M., MacDonald P.D., Stewart J.R. (2014). A case control study of environmental and occupational exposures associated with methicillin resistant *Staphylococcus aureus* nasal carriage in patients admitted to a rural tertiary care hospital in a high density swine region. Environ. Health.

[40] Monaco M., Pedroni P., Sanchini A., Bonomini A., Indelicato A., Pantosti A. (2013). Livestock-associated methicillin-resistant *Staphylococcus aureus* responsible for human colonization and infection in an area of Italy with high density of pig farming. BMC Infect. Dis..

[41] Anker J.C.H., Koch A., Ethelberg S., Molbak K., Larsen J., Jepsen M.R. (2018). Distance to pig farms as risk factor for community-onset livestock-associated MRSA CC398 infection in persons without known contact to pig farms-A nationwide study. Zoonoses Public Health.

[42] Lewis H.C., Molbak K., Reese C., Aarestrup F.M., Selchau M., Sorum M., Skov R.L. (2008). Pigs as source of methicillin-resistant *Staphylococcus aureus* CC398 infections in humans, Denmark. Emerg. Infect. Dis..

[43] Smith T.C., Gebreyes W.A., Abley M.J., Harper A.L., Forshey B.M., Male M.J., Martin H.W., Molla B.Z., Sreevatsan S., Thakur S. (2013). Methicillin-resistant *Staphylococcus aureus* in pigs and farm workers on conventional and antibiotic-free swine farms in the USA. PLoS ONE.

[44] Sorensen A.I.V., Jensen V.F., Boklund A., Halasa T., Christensen H., Toft N. (2018). Risk factors for the occurrence of livestock-associated methicillin-resistant *Staphylococcus aureus* (LA-MRSA) in Danish pig herds. Prev. Vet. Med..

[45] Yano T., Premashthira S., Dejyong T., Tangtrongsup S., Salman M.D. (2018). The effectiveness of a foot and mouth disease outbreak control programme in Thailand 2008–2015: Case studies and lessons learned. Vet. Sci..

[46] Vikram A., Bomberger J.M., Bibby K.J. (2015). Efflux as a glutaraldehyde resistance mechanism in *Pseudomonas fluorescens* and *Pseudomonas aeruginosa* biofilms. Antimicrob. Agents Chemother..

[47] Huang L., Wang M., Mo T., Liu M., Biville F., Zhu D., Jia R., Chen S., Zhao X., Yang Q. (2019). Role of *LptD* in resistance to glutaraldehyde and pathogenicity in Riemerella anatipestifer. Front. Microbiol..

[48] Kampf G. (2019). Antibiotic resistance can be enhanced in gram-positive species by some biocidal agents used for disinfection. Antibiotics.

[49] Kumari D.N., Keer V., Hawkey P.M., Parnell P., Joseph N., Richardson J.F., Cookson B. (1997). Comparison and application of ribosome spacer DNA amplicon polymorphisms and pulsed-field gel electrophoresis for differentiation of methicillin-resistant *Staphylococcus aureus* strains. J. Clin. Microbiol..

[50] Al-Talib H., Yean C.Y., Al-Khateeb A., Hassan H., Singh K.K., Al-Jashamy K., Ravichandran M. (2009). A pentaplex PCR assay for the rapid detection of methicillin-resistant *Staphylococcus aureus* and Panton-Valentine Leucocidin. BMC Microbiol..

[51] Fang H., Hedin G. (2003). Rapid screening and identification of methicillin-resistant *Staphylococcus aureus* from clinical samples by selective-broth and real-time PCR assay. J. Clin. Microbiol..

[52] van Doornum G.J., Guldemeester J., Osterhaus A.D., Niesters H.G. (2003). Diagnosing herpesvirus infections by real-time amplification and rapid culture. J. Clin. Microbiol..

[53] R Core Team R: A Language and Environment for Statistical Computing.

