# Supplementary material for: Acquisition Risk Factors of the SCCmec IX-Methicillin-Resistant Staphylococcus aureus in Swine Production Personnel in Chiang Mai and Lamphun Provinces, Thailand
Source: Antibiotics (Basel). 2020 Sep 29;9(10):651. doi: 10.3390/antibiotics9100651 (PMC7601853; doi:10.3390/antibiotics9100651)
Supplement: Supplementary file 1 [file antibiotics-09-00651-s001.pdf]

Acquisition Risk Factors of the SCC*mec* IX- Methicillin-resistant *Staphylococcus aureus* in Swine Production Personnel in Chiang Mai and Lamphun Provinces, Thailand

Peerapat Rongsanam [1], Terdsak Yano [2], Wuttipong Yokart [3], Panuwat Yamsakul [2], Suweera Suthammeng [1], Ratchadaporn Udpuan [1], Duangporn Pichpol [4], Decha Tamdee [5] and Usanee Anukool [1,6,*]

1. Division of Clinical Microbiology, Department of Medical Technology, Faculty of Associated Medical Sciences, Chiang Mai University, Chiang Mai, 50200, Thailand
2. Department of Food Animal Clinic, Faculty of Veterinary Medicine, Chiang Mai University, Chiang Mai, 50100, Thailand
3. Division of Clinical Microscopy, Department of Medical Technology, Faculty of Associated Medical Sciences, Chiang Mai University, Chiang Mai, 50200, Thailand
4. Department of Veterinary Biosciences and Veterinary Public Health, Faculty of Veterinary Medicine, Chiang Mai University, Chiang Mai, 50100, Thailand
5. Department of Public Health Nursing, Faculty of Nursing, Chiang Mai University, Chiang Mai, 50200, Thailand
6. Infectious Diseases Research Unit (IDRU), Faculty of Associated Medical Sciences, Chiang Mai University, Muang District, Chiang Mai, 50200, Thailand

* Correspondence: usanee.anukool@cmu.ac.th; Tel.: +66-53-93-5068 ext. 15, +66-86-654-4565; Fax: +66-53-93-6042

Supplementary Data

Table S1. The sampling sites, locations and sample code of 202 swine production personnel.

Sampling sites*	Locations	No. of SPP (SPP code)
A	Muang district, Lamphun	4 (A1, A2, A3, A4)
B	Muang district, Lamphun	4 (B1, B2, B3, B4)
C	Muang district, Lamphun	2 (C1, C2)
D	Muang district, Lamphun	5 (D1, D2, D3, D4, D5)
E	Muang district, Lamphun	3 (E1, E2, E3)
F	Muang district, Lamphun	1 (F1)
G	Muang district, Lamphun	1 (G1)
H	Muang district, Lamphun	1 (H1)
I	Muang district, Lamphun	2 (I1, I2)
J	Muang district, Lamphun	2 (J1, J2)
K	Muang district, Lamphun	1 (K1)
L	Muang district, Lamphun	2 (L1, L2)
M	Muang district, Lamphun	1 (M1)
N	Muang district, Lamphun	1 (N1)
O	Muang district, Lamphun	1 (O1)
P	Muang district, Lamphun	6 (P1, P2, P3, P4, P5, P6)

Q	Muang district, Lamphun	1 (Q1)
R	Muang district, Lamphun	1 (R1)
S	Muang district, Lamphun	1 (S1)
T	Muang district, Lamphun	7 (T1, T2, T3, T4, T5, T6, T7)
U	Mae Rim district, Chiang Mai	6 (U1, U2, U3, U4, U5, U6)
V	Mae Rim district, Chiang Mai	2 (V1, V2)
W	San Sai district, Chiang Mai	46 (W1, W2, W3, W4, W5, W6, W7, W8, W9, W10, W11, W12, W13, W14, W15, W16, W17, W18, W19, W20, W21, W22, W23, W24, W25, W26, W27, W28, W29, W30, W31, W32, W33, W34, W35, W36, W37, W38, W39, W40, W41, W42, W43, W44, W45, W46)
X	Muang district, Lamphun	14 (X1, X2, X3, X4, X5, X6, X7, X8, X9, X10, X11, X12, X13, X14)
Y	Muang district, Lamphun	4 (Y1, Y2, Y3, Y4)
Z	Pa Sang district, Lamphun	2 (Z1, Z2)
AA	Chom Thong district, Chiang Mai	41 (AA1, AA2, AA3, AA4, AA5, AA6, AA7, AA8, AA9, AA10, AA11, AA12, AA13, AA14, AA15, AA16, AA17, AA18, AA19, AA20, AA21, AA22, AA23, AA24, AA25, AA26, AA27, AA28, AA29, AA30, AA31, AA32, AA33, AA34, AA35, AA36, AA37, AA38, AA39, AA40, AA41)
AB	Muang district, Lamphun	4 (AB1, AB2, AB3, AB4)
AC	Chom Thong district, Chiang Mai	9 (AC1, AC2, AC3, AC4, AC5, AC6, AC7, AC8, AC9)
AD	Muang district, Lamphun	2 (AD1, AD2)
AE	Muang district, Lamphun	1 (AE1)
AF†	Muang district, Chiang Mai	21 (AF1, AF2, AF3, AF4, AF5, AF6, AF7, AF8, AF9, AF10, AF11, AF12, AF13, AF14, AF15, AF16, AF17, AF18, AF19, AF20, AF21)
AG‡	Muang district, Lamphun	1 (AG1)
Total		202

No., Number; SPP, swine production personnel

- A-AE: swine farms A-AE

† AF: the group of veterinarians and veterinary students who not routinely work at particular swine farm

‡ AG: the group of workers who was working at the slaughterhouse

Table S2. The accumulated resistance of 59 MRSA isolates from SPP.

No. of antimicrobial classes	Accumulated Resistance	
	No. of MRSA isolates	Percentage
10	1	1.69
9	8	13.56
8	26	44.07
7	43	72.88
6	48	81.36
5	55	93.22
4	56	94.92
3	59	100.00

© 2020 by the authors. Licensee MDPI, Basel, Switzerland. This article is an open access article distributed under the terms and conditions of the Creative Commons Attribution (CC BY) license (http://creativecommons.org/licenses/by/4.0/).
